# Upregulation of miR-501-5p activates the wnt/β-catenin signaling pathway and enhances stem cell-like phenotype in gastric cancer

**DOI:** 10.1186/s13046-016-0432-x

**Published:** 2016-11-15

**Authors:** Dongmei Fan, Baoqi Ren, Xiaojun Yang, Jia Liu, Zhengzheng Zhang

**Affiliations:** 1 Department of Gastroenterology, First Affiliated Hospital of Guangzhou University of Chinese Medicine, Guangzhou, 510405 China; 2 Medical Department of Guangdong Hospital of Traditional Chinese Medicine, Guangzhou, 510405 China; 3 Third Affiliated Hospital of Guangzhou Medical College, Guangzhou, China

**Keywords:** miR-501-5p, Gastric cancer, Wnt/β-catenin signaling, Cancer stem cell

## Abstract

**Background:**

miRNAs are critical post-transcriptional regulators of gene expression and key mediators of tumourigenesis. miR-501-5p is newly identified to be involved in the tumor progression, but its biological role and mechanism remain largely unknown. This study is aimed to study the role of miR-501-5p in the progression of gastric cancer.

**Methods:**

Real-time PCR analysis was used to determine miR-501-5p expression in gastric cancer cell lines, clinical tissues and 112 clinicopathologically characterized gastric cancer specimens. The role of miR-501-5p in maintaining gastric cancer stem cell like phenotype was examined by tumor-sphere formation assay and expression of stem cell markers. Luciferase reporter assay, cellular fractionation and western blot analysis were used to determined that miR-501-5p activated the wnt/β-catenin signaling by directly targeting DKK1, NKD1 and GSK3β.

**Results:**

Herein, our results revealed that miR-501-5p was markedly upregulated in gastric cancer cell lines and clinical tissues. High miR-501-5p levels predicted poor overall survival in gastric cancer patients. Gain-of-function and loss-of-function studies showed that ectopic expression of miR-501-5p enhanced the cancer stem cell-like phenotype in gastric cancer cells. Notably,wnt/β-catenin signaling was hyperactivated in gastric cancer cells that overexpress miR-501-5p, and mediated miR-501-5p-induced cancer stem cell-like phenotype. Furthermore, miR-501-5p directly targeted and suppressed multiple repressors of the wnt/β-catenin signaling cascade, including DKK1, NKD1 and GSK3β. These results demonstrate that miR-501-5p maintains constitutively activated wnt/β-catenin signaling by directly targeting DKK1, NKD1 and GSK3β, which promotes gastric cancer stem cell like phenotype.

**Conclusions:**

Taken together, our findings reveal a new regulatory mechanism of miR-501-5p and suggest that miR-501-5p might be a potential target in gastric cancer therapy.

**Electronic supplementary material:**

The online version of this article (doi:10.1186/s13046-016-0432-x) contains supplementary material, which is available to authorized users.

## Background

Gastric cancer (GC) is the fifth most common malignancy and the third leading cause of cancer-related death worldwide. It is estimated that 951,600 new GC cases and 723,100 deaths occurred in 2012 [1]. In most countries, survival from stomach cancer remained in the narrow range of 25–30% [2]. Although therapeutic methods are improving in surgical combined with radiotherapy and chemotherapy, the prognosis for advanced stage patients is still very poor [3, 4]. Recent studies have revealed that gastric cancer stem cells constitute a reservoir of selfsustaining cells with the exclusive ability to self-renew and cause tumor outgrowth [5].^.^ Integral to the CSC model is a subpopulation at the apex of the hierarchy (usually comprising <5% of the cancer), responsible for the formation, maintenance, and continued growth of the tumour [6]. However, the molecular mechanisms for the maintenance of GC stem cell phenotype remains largely unknown.

The Wnt/β-catenin signaling pathway is considered as a key player in the regulation of tissue homeostasis, organ size, tumorigenesis [7–9]. Upon activation, the wnt signals will stabilize and finally lead to accumulation of β-catenin. Activated β-catenin dissociates with E-cadherin, dissembling the adherens and enters the nucleus to turn on the expression of target genes, most of which show invasion promotion functions [10]. Notably, recent advances have shown that the wnt/β-catenin signaling pathway is involved in the maintenance of the GC stem cell population [11, 12]. It has been reported that the pathway blocked by dickkopf WNT signaling pathway inhibitor 1 (DKK-1), naked cuticle homolog 1 (NKD1) and glycogen synthase kinase 3 beta (GSK3β) caused a robust reduction in the activity of wnt/β-catenin signaling and self-renewing capacity of gastric cancer cells [12–16]. Accordingly, the wnt/β-catenin signaling pathway is considered as an important regulator in the maintenance of CSC population, and better understanding of the mechanisms that regulate wnt/β-catenin pathway may provide new clues for more effective GC therapy.

MicroRNAs (miRNAs) are a class of highly conserved, small noncoding RNAs that are approximately 22 nucleotides in length. Typically, miRNAs bind to the 3′-untranslated regions (3′-UTRs) of mRNAs, guide the formation of miRNA-mRNA-induced silencing complexes and lead to the degradation or inhibit the translation of the targeted mRNAs [17, 18]. Bioinformatic predictions indicate that miRNAs regulate more than 30 % of the protein-coding genes [19]. The dysregulation of miRNA expression appears to be a general trait of GC [20]. Thus, it is of particular interest to identify miRNAs that might interfere with wnt/β-catenin signaling and thereby lead to the self-renewal of GC stem cells.

In the present study, we sought to elucidate the the effect of miR-501-5p on wnt/β-catenin signaling in GC, as well as the related molecular mechanism by which miR-501-5p affected wnt/β-catenin signaling. Our data showed that miR-501-5p was upregulated in GC tissues and correlated with a more aggressive phenotype of GC in patients. miR-501-5p activates the wnt/β-catenin pathway by directly targeting DKK1, NKD1 and GSK3β, and consequently enhances stem cell-like phenotype of GC.

## Methods

### Tissues and cells

Fresh GC tissue samples from GC patients, and their matched adjacent non-tumor gastric mucosal tissues (>5 cm laterally from the edge of tumor region) were obtained from the First Affiliated Hospital of Guangzhou University of Chinese Medicine. The samples had been clinically and histopathologically diagnosed according to the World Health Organization criteria. Tumor and non-cancerous tissues were confirmed histologically by hematoxylin and eosin staining. All samples were collected from consenting individuals according to the protocols approved by the Ethics Review Board at Guangzhou University of Chinese Medicine. GC cell lines SGC-7901, HGC-27, MGC-803, MKN-28 and BGC-823 were routinely maintained in DMEM medium (Invitrogen, Carlsbad, CA) supplemented with 10% fetal bovine serum (HyClone, Logan, UT).

### Plasmids and oligonucleotides

The TOP/FOP Luciferase Reporting system was purchased from lifeome (Oceanside, CA, USA). miR-501-5p and miR-501-5p antagonist (antagomiR-501-5p, RiboBio, Guangzhou, China) were used to overexpress or inhibit miR-501-5p. The 3′UTRs of human DKK1, NKD1 and GSK3β were generated by PCR amplification and subcloned into the pGL3 vector plasmid (Promega, Madison, WI). The primers are as the following:

- DKK1-3′UTR-luc-up, 5′-GCCCCGCGGCACTAAACCAGCTATCCA-3′;
- DKK1-3′UTR-luc-dn, 5′-GCCCTGCAGTAGGCAGTGCAGCACCTT-3′;
- NKD1-3′UTR-luc-up, 5′-GCCCCGCGGGAGCCTGGAGAAACCTGAAA-3′;
- NKD1-3′UTR-luc-dn, 5′-GCCCTGCAGCCCACATCAACAAGCTCCCT-3′;
- GSK3B-3′UTR-luc-up, 5′-GCCCCGCGGTGCCTCAAAGTAGTCCAT-3′;
- GSK3B-3′UTR-luc-dn, 5′-GCCCTGCAGGTGTTTGGCTCTGTGATT-3′;

The siRNA sequences were as followings:

- β-catenin-siRNA: 5′-CCAUUGUUUGUGCAGCUGCUU-3′;
- DKK1-siRNAi: 5′-ACACUUGUCAGAGACACUAAA-3′;
- NKD1-siRNAi: 5′-CCACUUAAACAAGCGUGGUUU-3′;
- GSK3B-siRNAi: 5′-CCACUCAAGAACUGUCAAGUA-3′.

### Western blot analysis

Cells were harvested in cell lysis buffer (Cell Signaling Technology; Cat#: 9803) and heated for 5 min at 100 °C. Equal quantities of denatured protein samples were resolved on 10% SDS-polyacrylamide gels and then transferred onto polyvinylidene difluoride membranes (Roche). After blocking with 5% non-fat dry milk in Tris-buffered saline/0.05% Tween 20 (TBST), the membrane was incubated with a specific primary antibody followed by a horseradish peroxidase-conjugated secondary antibody. Proteins were visualized using ECL reagents (Pierce). The antibodies used were as follows: anti-β-catenin, anti-DKK1, anti-NKD1 and anti-GSK3β (Abcam, Cambridge, MA, USA), and p-GSK3β (Ser9) (Cell Signaling Technology). The membranes were stripped and reprobed with an anti–α-tubulin antibody (Sigma-Aldrich, St. Louis, MO, USA) as the loading control.

### MiRNA extraction and real-time quantitative PCR

Total miRNA from cultured cells and fresh surgical gastric cancer tissues was extracted using a mirVana miRNA Isolation Kit (Ambion, Austin, TX, USA) according to the manufacturer’s instructions. We synthesised cDNA from 10 ng total RNA using a TaqMan miRNA reverse transcription kit (Applied Biosystems, Foster City, CA, USA), and quantified the expression levels of miR-501-5p using a miRNA-specific TaqMan MiRNA Assay Kit (Applied Biosystems). miRNA expression was defined based on the threshold cycle (Ct), and relative expression levels were calculated as 2^-[(Ct of^ ^miR-501-5p) – (Ct of U6)]^ after normalization with reference to expression of U6 small nuclear RNA.

### Sphere formation assays

1 × 10^3^ cells were seeded in 6-well ultra low cluster plates (Corning, NY) and about 10 cells were seeded in 24-well ultra low cluster plates (Corning, NY) for 15 days. Spheres were cultured in DMEM/F12 serum-free medium (Invitrogen, Grand Island, NY) supplemented with 2% B27 (Invitrogen, Grand Island, NY), 20 ng/ml of EGF, and 20 ng/ml of bFGF (PeproTech, Offenbach, Germany), 0.4% bovine serum albumin (BSA) (Sigma, St. Louis, MO, USA), and 5 μg/ml insulin.

### Luciferase assays

Cells (4 × 10^4^) were seeded in triplicate in 24-well plates and cultured for 24 h. Cells were transfected with 100 ng TOP/FOP reporter luciferase plasmid, or pGL3-DKK1-3′UTR, pGL3-NKD1-3′UTRor pGL3-GSK3B-3′UTR luciferase plasmids, plus 5 ng pRL-TK Renilla plasmid (Promega) using Lipofectamine 2000 (Invitrogen) according to the manufacturer’s recommendation. Luciferase and Renilla signals were measured 36 h after transfection using a Dual Luciferase Reporter Assay Kit (Promega) according to the manufacturer’s protocol.

### Statistical analyses

All statistical analyses were carried out using SPSS statistical software (SPSS Inc., Chicago, IL, USA). The 2-tailed Student’s t-test was used to evaluate the significance of the differences between two groups of data in all pertinent experiments; a *P* value <0.05 was considered significant.

## Results

### miR-501-5p is upregulated in human gastric cancer tissues and cell lines

**Fig. 1:**
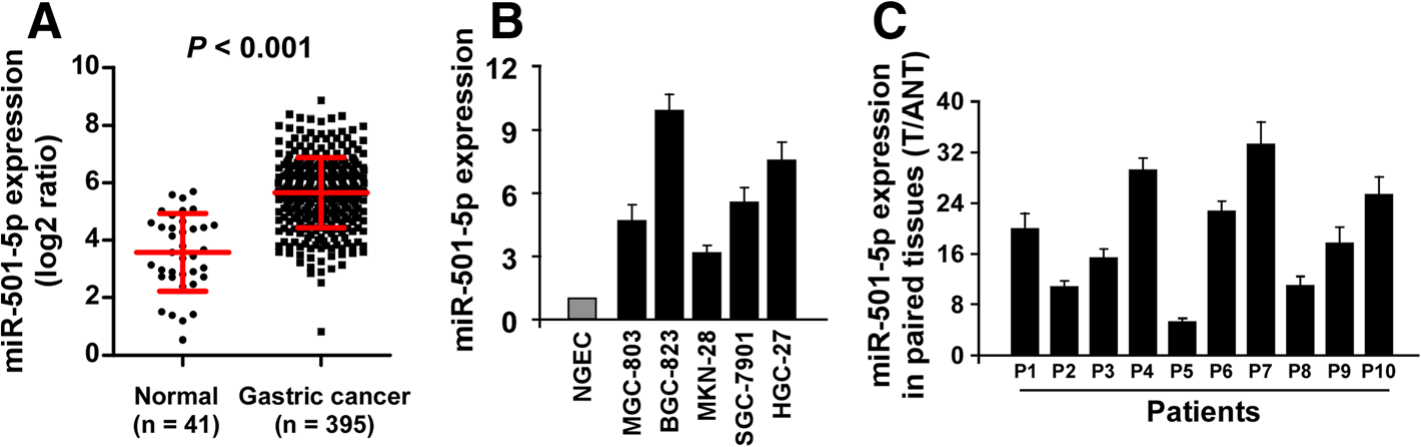
miR-501-5p is upregulated in human gastric cancer tissues and cell lines. **a** miR-501-5p levels remained low in normal gastric tissues but became dramatically elevated in gastric cancer tissues by analyzing The Cancer Genome Atlas (TCGA) gastric cancer miRNA sequencing data sets (Normal, *n* = 41; gastric cancer, *n* = 395). *P* < 0.001, 2-tailed Student’s *t*-test. **b** and **c** Real-time PCR analysis of miR-501-5p expression in normal gastric epithelial cell (NGEC) and 5 cultured gastric cancer cell lines (**b**), and in 10 pairs of gastric cancer samples (T) and adjacent normal tissues (ANT) (**c**). Transcript levels were normalized by *U6* expression. Error bars represent the mean ± s.d. of three independent experiments. *P < 0.05

By analysis of the The Cancer Genome Atlas (TCGA) Stomach adenocarcinoma miRNA sequencing data sets, we found that miR-501-5p levels were significantly upregulated in human gastric cancer tissues (*n* = 395) compared with that in normal gastric tissues (*n* = 41) (*p* < 0.001) (Fig. 1a). We further verified this result in gastric cancer cell lines and paired tissues by real-time PCR analysis. As shown in Fig. 1b and c, miR-501-5p levels were differentially increased in 5 gastric cancer cell lines than that in normal gastric epithelial cell (NGEC), and in 10 gastric cancer tissues (T) compared to that in the paired adjacent normal tissues (ANT). Collectively, these results suggest that miR-501-5p is upregulated and might be involved in human gastric cancer progression.

### High miR-501-5p predicts poor prognosis

**Fig. 2:**
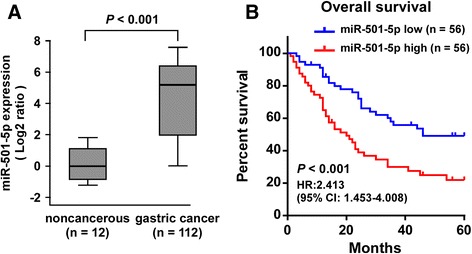
High miR-501-5p predicts poor prognosis*.* **a** Real-time PCR analysis of miR-501-5p expression in 112 freshly collected gastric cancer tissues compared to that in 12 non-cancerous gastric tissues. Transcript levels were normalized to *U6* expression. Boundaries of boxes represent lower and upper quartiles, respectively. Lines within boxes and whiskers denote median and extremum, respectively. *P* < 0.001, 2-tailed Student’s *t*-test. **b** Kaplan–Meier analysis of 5-year overall survival curves of patients with gastric cancer with high miR-501-5p expression (> median, *n* = 56) versus low miR-501-5p expression (< median, *n* = 56) [hazard ratio (HR) = 2.413; 95% confidence interval (CI): 1.453–4.008]. *P* < 0.001, log-rank test

We further assessed the clinical significance of miR-501-5p expression levels in 112 gastric cancer tissues. As shown in Fig. 2a, miR-501-5p expression was markedly increased in 112 gastric cancer samples compared with that in 12 non-cancerous gastric tissues. Importantly, patients with high miR-501-5p expression had a significant poor overall survival compared to patients with low miR-501-5p expression (*P* < 0.001; hazard ratio = 2.413, 95% CI = 1.453–4.008; Fig. 2b).

### miR-501-5p enhances the stem cell-like phenotype in gastric cancer cells

**Fig. 3:**
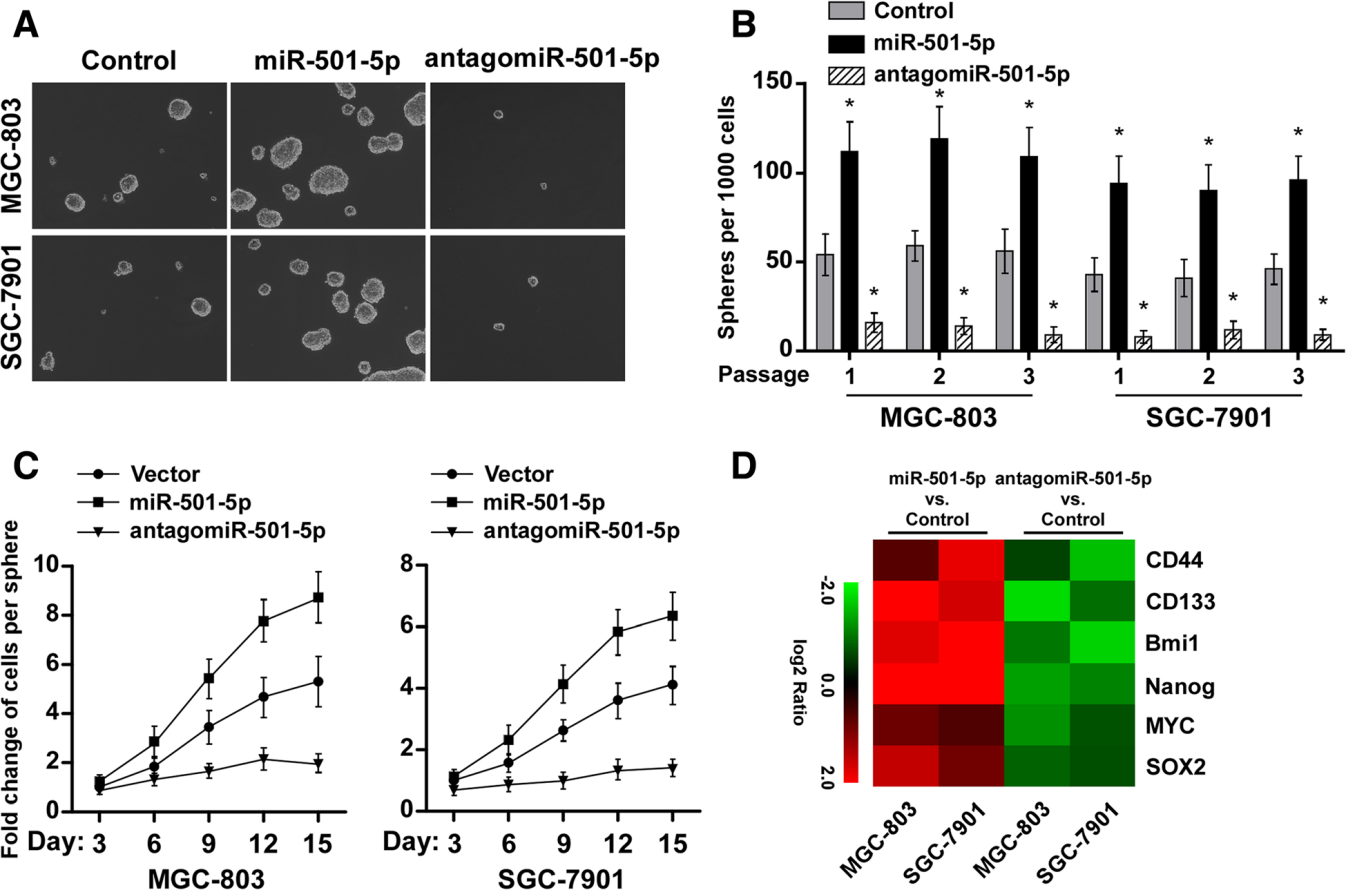
miR-501-5p enhances the stem cell-like phenotype in gastric cancer cells. **a** Representative micrographs of tumor spheres formed by indicated cells. **b** Histograms showing the mean number of spheres formed by the indicated cells from different passages. **c** The fold change in the number of cells per sphere on the indicated days. **d** Real-time PCR analysis revealed that miR-501-5p regulates the expression levels of numerous stem cell regulators. The pseudocolors represent the intensity scale of expression in miR-501-5p vs. control-transfected cells or antagomiR-501-5p vs. control-transfected cells generated by log_2_ transformation. Error bars represent the mean ± s.d. of three independent experiments. * *P* < 0.05

It has been noted that aquisition of stem cell-like phenotype of gastric cancer cells is crucial for the malignance and high frequency of relapse [5]. We then investigated the role of miR-501-5p upregulation in the self-renewal ability of gastric cancer cells. Gastric cancer cell lines MGC-803 and SGC-7901 were engineered to overexpress or silence miR-501-5p by transfection of miR-501-5p mimic or antagomiR-501-5p (Additional file 1: Figure S1). Notably, overexpression of miR-501-5p robustly promoted gastric cancer cells cultured in suspension to generate approximately two-fold more tumor spheres and higher cell content, compared with the spheres formed by control cells until day 15 (Fig. 3a–c). Conversely, miR-501-5p-silenced cells formed ~3-fold fewer spheres and lower cell content, compared with control cells (Fig. 3a–c). Moreover, flow cytometry assays revealed a higher percentage of the side-population of cells in the miR-501-5p overexpressing cells but a lower percentage in the miR-501-5p-inhibited cells compared to control (Additional file 2: Figure S2). In addition, overexpression of miR-501-5p increased, while downregulation of miR-501-5p reduced the expression levels of different stem cell regulators including CD44, CD133, Bmi1, Nanog, MYC and SOX2 (Fig. 3d). Thus, our results indicate that miR-501-5p promotes the stem cell-like phenotype in gastric cancer.

### miR-501-5p activates wnt/β-catenin signaling pathway

**Fig. 4:**
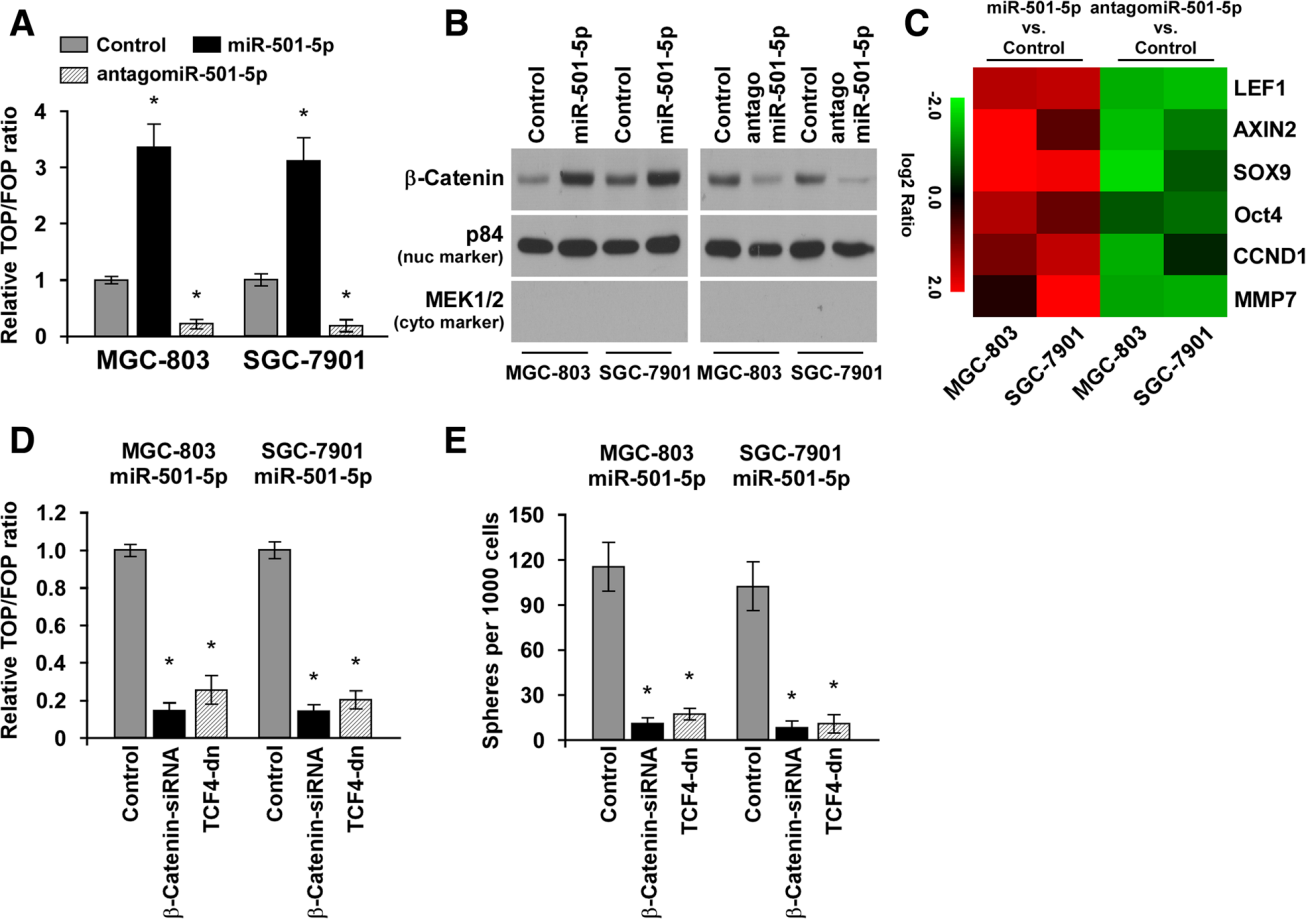
miR-501-5p activates wnt/β-catenin signaling pathway. **a** TOP/FOP luciferase reporter assays in indicated cells. **b** Western blotting analysis of β-catenin in nuclear fraction of cells. The nuclear protein p84 was used as the nuclear protein marker. MEK1/2 was used as the cytoplasmic marker. **c** Real-time PCR analysis revealed that miR-501-5p regulates the expression levels of multiple wnt/β-catenin downstream genes in gastric cancer cells. The pseudocolors represent the intensity scale of expression in miR-501-5p vs. control cells, or antagomiR-501-5p vs. control cells generated by log_2_ transformation. **d** The stimulatory effect of miR-501-5p on TOP/FOP luciferase reporter activity was impaired by silencing β-catenin or expressing TCF4-dn. **e** Sphere formation assays indicated that silencing β-catenin or expressing TCF4-dn abrogated the promotive effects of miR-501-5p on self-renewal of gastric cancer cells. Error bars represent the mean ± s.d. of three independent experiments. *P < 0.05

Since wnt/β-catenin signaling is one of the most important pathways in maintaining stem cell phenotype and frequently activated in gastric cancer, we then examined the role of miR-501-5p in wnt/β-catenin signaling pathway. As shown in Fig. 4a, we found that miR-501-5p overexpression significantly increased, but silencing of miR-501-5p reduced the TOP/FOP luciferase reporter activity. In addition, cellular fractionation and western blot analysis revealed that overexpression of miR-501-5p increased nuclear accumulation of β-catenin, while silencing of miR-501-5p reduced nuclear β-catenin expression (Fig. 4b). Moreover, real-time PCR analysis revealed that miR-501-5p upregulated, but downregulation of miR-501-5p repressed the expression levels of the multiple wnt/β-catenin downstream genes (Fig. 4c). Collectively, our results suggest that miR-501-5p activates Wnt/β-catenin signaling pathway in gastric cancer.

We further investigated the functional significance of wnt/β-catenin signaling activation in miR-501-5p–mediated self-renewal of gastric cancer cells by silencing β-catenin or expressing TCF4-dn in miR-501-5p–overexpressing MGC-803 and SGC-7901 cells. As expected, the stimulatory effect of miR-501-5p on TOP/FOP luciferase reporter activity was impaired by silencing β-catenin or expressing TCF4-dn (Fig. 4d). Moreover, sphere formation assays indicated that silencing β-catenin or expressing TCF4-dn abrogated the promotive effects of miR-501-5p on self-renewal of gastric cancer cells (Fig. 4e). Thus, these results reveal that activation of wnt/β-catenin signaling is essential for miR-501-5p–promoted stem cell like phenotype in gastric cancer.

### miR-501-5p directly targets multiple repressors of wnt/β-catenin signaling pathway

**Fig. 5:**
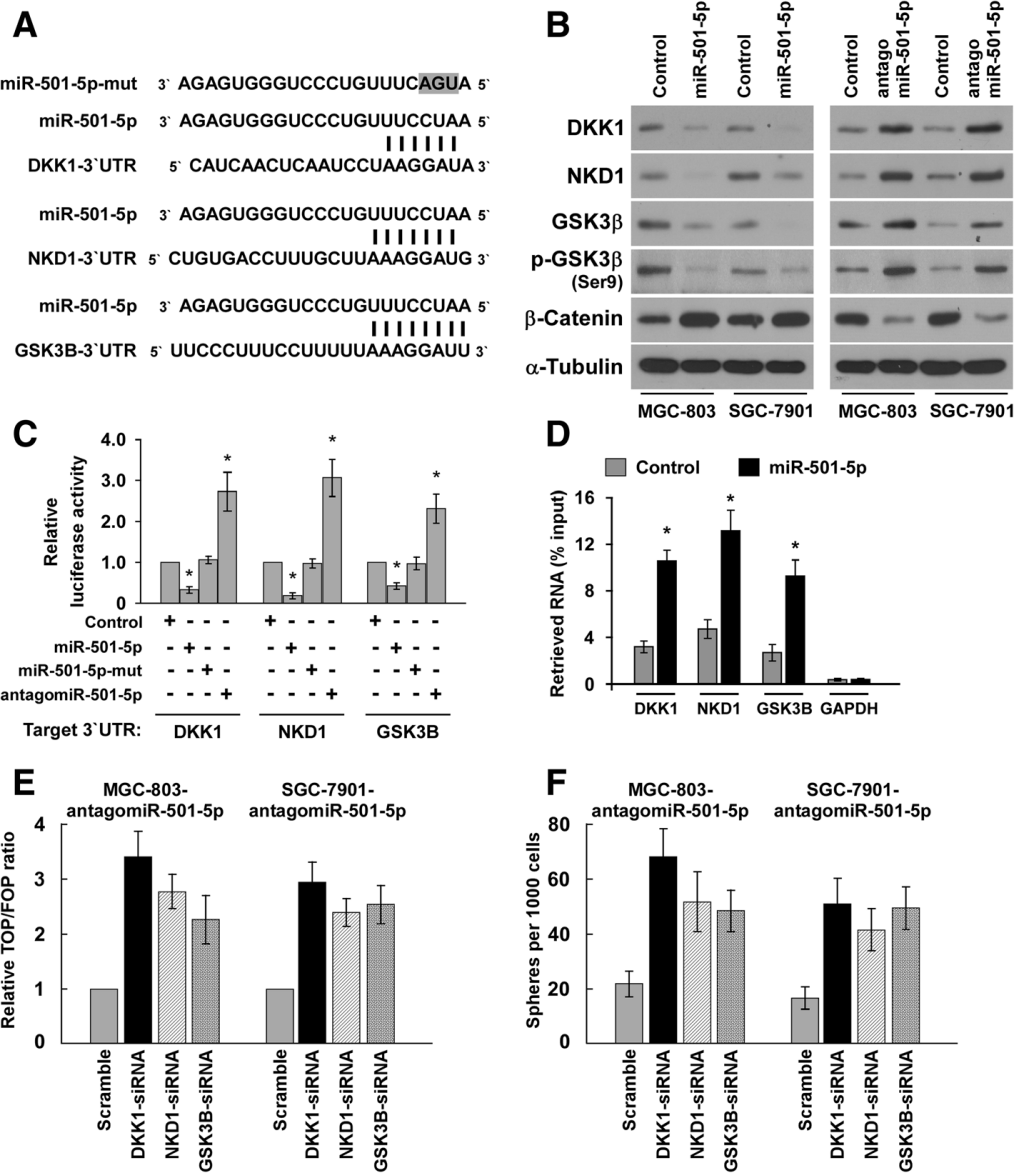
miR-501-5p directly targets multiple repressors of wnt/β-catenin signaling pathway. **a** Predicted miR-501-5p target sequence in 3'UTRs of DKK1, NKD1 and GSK3B. The mutated miR-501-5p containing three altered nucleotides were indicated. **b** Western blots of DKK1, NKD1, GSK3β, p-GSK3β (Ser9) and β-catenin expression. α-Tubulin served as the loading control. **c** Luciferase assay of pGL3-DKK1-3′UTR, pGL3-NKD1-3'UTR or pGL3-GSK3B-3'UTR reporter in the miR-501-5p-, antagomiR-501-5p-, mutant miR-501-5p- and control-transfected MGC-803 cells. **d** MiRNP IP assay showing the association between miR-501-5p and DKK1, NKD1 and GSK3B transcripts in MGC-803 cells. *GAPDH* served as the negative control. **e** Individual silencing of DKK1, NKD1 and GSK3B potently rescued the TOP/FOP luciferase reporter activity and self-renewal ability in miR-501-5p-inhibited gastric cancer cells (Fig. 5**e** and **f**), demonstrating that these genes were functional effectors of miR-501-5p on regulating wnt/β-catenin signaling activation and stem cell-like phenotype in gastric cancer. Error bars represent the mean ± s.d. of three independent experiments. * *P* < 0.05

Interestingly, using the publicly available algorithms TargetScan and miRanda, we found that multiple repressors of wnt/β-catenin signaling, i.e., DKK1, NKD1 and GSK3B might be potential targets of miR-501-5p (Fig. 5a). Western blot analysis revealed that overexpression of miR-501-5p markedly reduced the expression levels of DKK1, NKD1 and GSK3β. In contrast, miR-501-5p inhibition increased them, suggesting that miR-501-5p negatively regulated these proteins (Fig. 5b). Meanwhile, overexpression of miR-501-5p increased, but inhibition of miR-501-5p repressed β-catenin expression in gastric cancer cells (Fig. 5b). Luciferase assay showed that miR-501-5p overexpression attenuated, while inhibition of miR-501-5p elevated the reporter activities driven by the 3′UTRs of DKK1, NKD1 and GSK3B transcripts (Fig. 5c). However, ectopic expression of the mutant miR-501-5p did not exhibit repressive effects on the reporter activities driven by the 3′UTRs of these transcripts (Fig. 5c). Moreover, microribonucleoprotein (miRNP) immunoprecipitation (IP) assay revealed a selective association of miR-501-5p with DKK1, NKD1 and GSK3B, but not with GAPDH (Fig. 5d), further indicating the specific effects of miR-501-5p on these targets. In addition, individual silencing of these targets potently rescued the TOP/FOP luciferase reporter activity and self-renewal ability in miR-501-5p-inhibited cells (Fig. 5e and f), demonstrating that DKK1, NKD1 and GSK3β were functional effectors of miR-501-5p on regulating wnt/β-catenin signaling activation and stem cell-like phenotype in gastric cancer. Collectively, our results suggest that miR-501-5p activates wnt/β-catenin signaling to enhance stem cell-like phenotype in gastric cancer by directly targeting DKK1, NKD1 and GSK3β.

## Discussion

MicroRNAs have been demonstrated to negatively regulate target mRNAs in a sequence-specific manner, and are key regulators in a wide variety of oncogenic processes, such as cell proliferation, differentiation, invasion and metastasis, functioning as either tumor suppressors or oncogenes [21–23]. Therefore, elucidating the underlying mechanism of miRNAs in tumor development may provide valuable diagnostic and therapeutic strategies for malignancy. Previous evidence has demonstrated that miR-501-5p is overexpressed in human hepatocellular carcinoma, and upregulation of miR-501-5p contributes to cancer cell proliferation, migration, invasion and drug resistance via different mechanisms [24]. Herein, combining with the TCGA gastric cancer microRNA data set analysis, our results revealed that miR-501-5p was markedly upregulated in GC tissues compared with paired adjacent normal tissues, and was significantly correlated with a more aggressive phenotype of GC in patients.

Gastric cancer continues to be a highly lethal malignancy, despite the use of multimodal treatment approaches. The CSC model has been proposed to explain the high rate of relapse and subsequent resistance of cancer to current systemic treatments [25]. CSCs have been identified in many solid malignancies, including gastric cancer, and have significant clinical implications, as targeting the CSC population may be essential in preventing the recurrence and spread of a tumour [26, 27]. Herein, we found that miR-501-5p acted as a potent CSC-promoting factor in GC. Overexpression of miR-501-5p promoted the self-renewal of GC cells and upregulated the pluripotency associated markers, including CD133, Bmi1, Nanog, MYC and Sox2. More importantly, inhibition of miR-501-5p by antagomiR-501-5p potentially reduced self-renewal of GC cells. Thus, our results suggest that upregulation of miR-501-5p in GC is involved in the malignant progression of GC, and propose that miR-501-5p might be a potential therapeutic target for human gastric cancer.

Accumulating evidence revealed that cancer stem cells have higher metastatic potency by inducing Epithelial–mesenchymal transition (EMT) [28]. Notably, Yoshida et al. recently found that oxidative stress-induced canonical Wnt activation play an important role in the heterogeneous cancer stem cell population at the invasive area by regulating CD44 and c-Myc [29]. Herein, we found that miR-501-5p increased the stemness in gastric cancer by activating the Wnt/β-catenin signaling and increasing the expression of CD44 and c-Myc. Thus, miR-501-5p might contribute to gastric cancer metastasis, and this hypothesis remains to be examined. For example, does miR-501-5p induce EMT; is miR-501-5p increased at the invasive edge of gastric cancer tissues; does miR-501-5p regulate the heterogeneous cancer stem cell population; these questions remain to be answer. Moreover, it was reported that Wnt activation could be induced by micro-environmental stress [29]. Therefore, it would be interesting to investigate whether micro-environmental stress is responsible for miR-501-5p upregulation in gastric cancer.

Several signaling pathways, including the wnt/β-catenin, Notch, and PI3K/Akt pathways, have been found to be aberrantly activated and play vital roles in the development and progression of gastric cancer [11, 30, 31] Among these pathways, the functions of wnt/β-catenin signaling in gastric cancer development and progression have been well documented. TCF4 and coactivator β-catenin are two key downstream effectors of the wnt/β-catenin signaling pathway, and considered as potent oncogenes in tumor progression [7]. Notably, unlike in other cancer types, the mutations in β-catenin are rare, but the expression and/or nuclear localization of β-catenin is often abnormal in gastric cancer, indicating a constitutive activation of Wnt/β-catenin signaling [7, 11, 12]. However, how cancer cells evade the negative regulation by wnt/β-catenin signaling cascade, leading to constitutively activated β-catenin/TCF remains unclear. Herein, we demonstrated that miR-501-5p was substantially overexpressed in gastric cancer and induced hyper-activation of β-catenin/TCF via directly targeting DKK1, NKD1 and GSK3β. Therefore, our findings not only suggest activation of Wnt/β-catenin pathway contributes to the malignant behavior of gastric cancer, but also reveal a novel mechanism for activation of wnt/β-catenin pathway involving miR-501-5p in gastric cancer.

It was reported that GSK-3β exhibited both oncogenic and tumor-suppressive roles in the context of different cancer types. For example, Tang et al. found that loss of GSK-3β expression activated β-Catenin to upregulate the expression of miR-183-96-182 cluster, leading to proliferation and migration of gastric cancer [32]. In contrast, inhibition of GSK3β compromises the survival, proliferation and invasion of glioblastoma cells [33]. Herein, we found that miR-501-5p repressed GSK3β to activate Wnt/β-Catenin pathway and enhance gastric cancer stem cell phenotype, further suggesting a tumor-repressive role of GSK3β in gastric cancer. Moreover, the role of miR-501-5p in glioblastoma remains to be further examined in future. Appropriate reference and discussion have been incorporated into revised manuscript.

## Conclusions

In conclusion, our study has revealed that miR-501-5p upregulation plays an important role in gastric cancer progression and miR-501-5p is a critical activator of Wnt/ β-catenin signaling by targeting DKK1, NKD1 and GSK3β. Understanding the precise role of miR-501-5p in gastric cancer pathogenesis and in the wnt/β-catenin signaling pathway promises to increase our knowledge of the biological basis of cancer development and may also facilitate the development of new therapeutic strategies against gastric cancer.

## Additional files

Additional file 1: Figure S1. Real-time PCR analysis of miR-501-5p in miR-501-5p-overexpressing, miR-501-5p-silenced and control MGC-803 and SGC-7901 cell lines. Transcript levels were normalized to U6 expression. (TIF 97 kb)
Additional file 2: Figure S2. Hoechst 33342 dye exclusion assay showing that overexpressing miR-501-5p promoted, whereas inhibition of miR-501-5p attenuated, the side-population cells in gastric cancer cells. (TIF 193 kb)
