# Supplementary figures and images for: Upregulation of miR-501-5p activates the wnt/β-catenin signaling pathway and enhances stem cell-like phenotype in gastric cancer
Source: J Exp Clin Cancer Res. 2016 Nov 15;35:177. doi: 10.1186/s13046-016-0432-x (PMC5111270; doi:10.1186/s13046-016-0432-x)

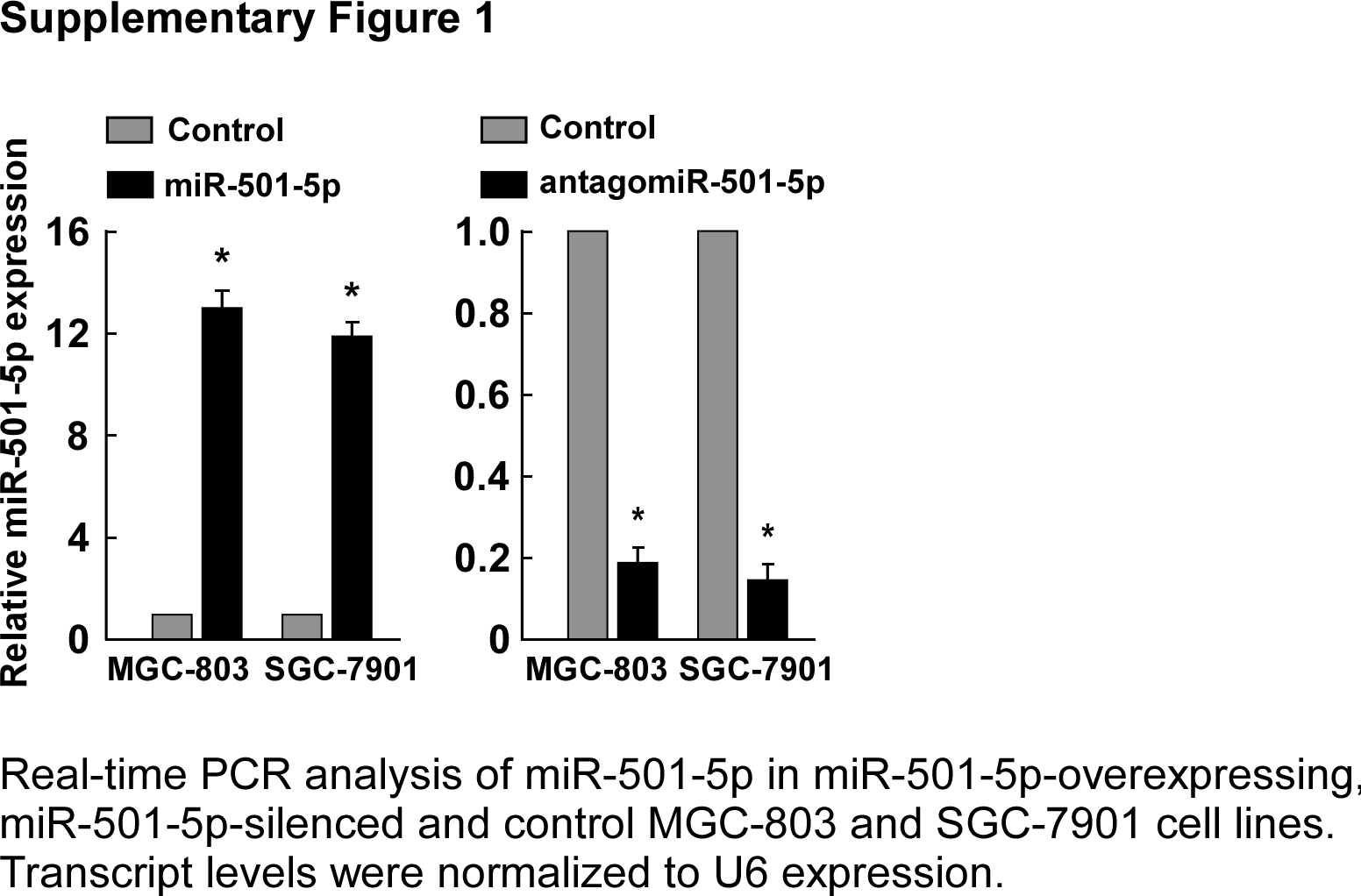

Supplement: Additional file 1: Figure S1. — Real-time PCR analysis of miR-501-5p in miR-501-5p-overexpressing, miR-501-5p-silenced and control MGC-803 and SGC-7901 cell lines. Transcript levels were normalized to U6 expression. (TIF 97 kb) [file 13046_2016_432_MOESM1_ESM.tif]

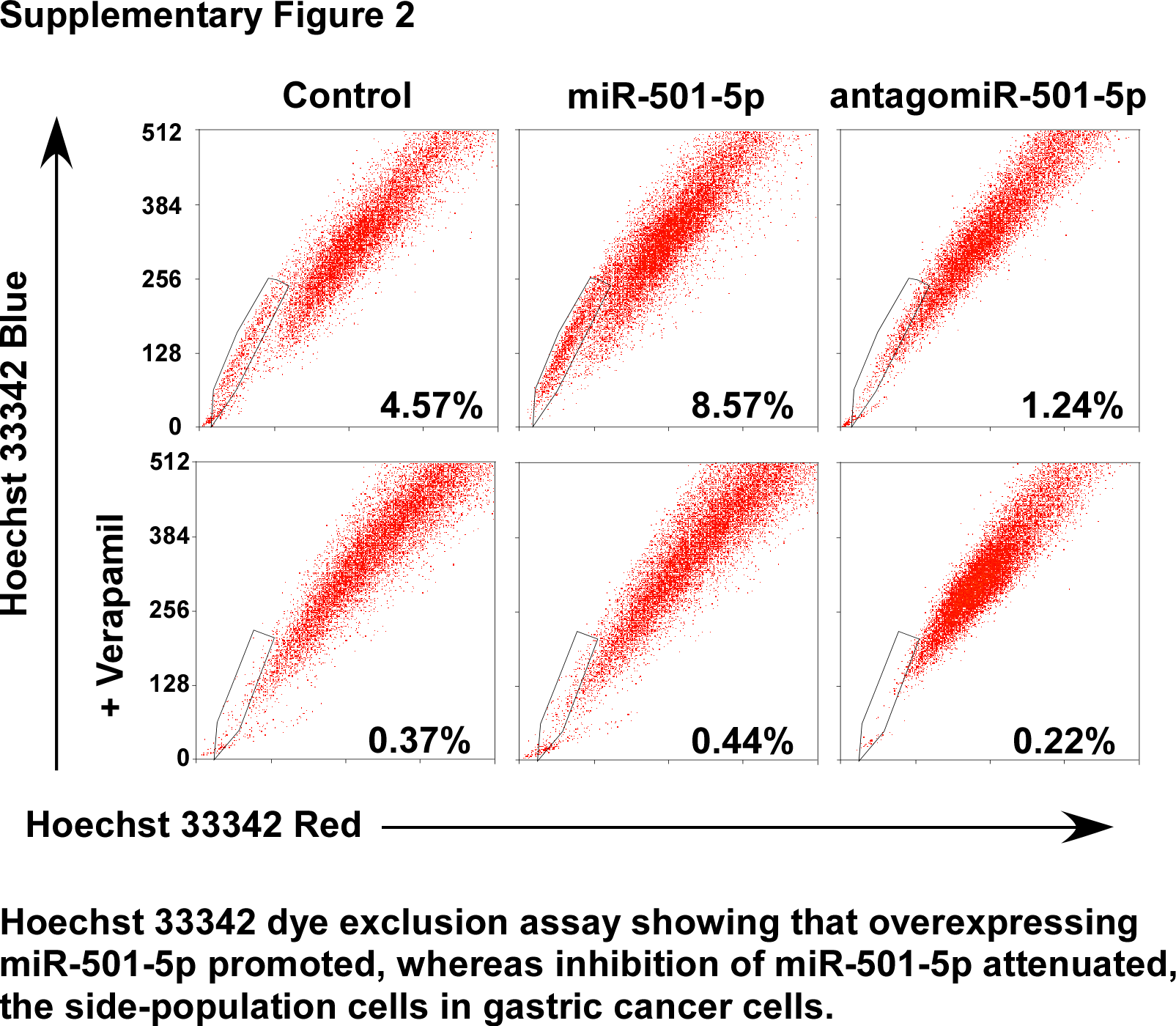

Supplement: Additional file 2: Figure S2. — Hoechst 33342 dye exclusion assay showing that overexpressing miR-501-5p promoted, whereas inhibition of miR-501-5p attenuated, the side-population cells in gastric cancer cells. (TIF 193 kb) [file 13046_2016_432_MOESM2_ESM.tif]
